# Bioprospecting Endophytic Fungi of Forest Plants for Bioactive Metabolites with Antibacterial, Antifungal, and Antioxidant Potentials

**DOI:** 10.3390/molecules29194746

**Published:** 2024-10-08

**Authors:** El-Sayed R. El-Sayed, Abirami Baskaran, Oliwia Pomarańska, Daria Mykhailova, Anna Dunal, Anita Dudek, Sahil Satam, Tomasz Strzała, Jacek Łyczko, Teresa Olejniczak, Filip Boratyński

**Affiliations:** 1 Department of Food Chemistry and Biocatalysis, Wrocław University of Environmental and Life Sciences, Norwida 25, 50-375 Wrocław, Poland; abirami.baskaran@upwr.edu.pl (A.B.); jacek.lyczko@upwr.edu.pl (J.Ł.); filip.boratynski@upwr.edu.pl (F.B.); 2 Plant Research Department, Nuclear Research Center, Egyptian Atomic Energy Authority, Cairo 11787, Egypt; 3 Department of Physics and Biophysics, Wrocław University of Environmental and Life Sciences, Norwida 25, 50-375 Wrocław, Poland; 4 Department of Genetics, Wrocław University of Environmental and Life Sciences, Ul. Kożuchowska 7, 51-631 Wrocław, Poland

**Keywords:** fungal endophytes, antifungal, antibacterial, antioxidant, gamma irradiation, forest trees, bioactivities

## Abstract

The growing emergence of multi-drug resistant microbial strains has kept the scientific world searching for novel bioactive compounds with specific chemical characteristics. Accordingly, researchers have started exploring the understudied metabolites from endophytes as a new source of bioactive compounds. In this context, the current study was designed to evaluate the bioactive properties of endophytic fungi from the Mokrzański forest in Wrocław, Poland that have not yet been fully researched. Forty-three endophytic fungi were isolated from twelve distinct plants. Following their cultivation, fungal extracts were separately prepared from biomass and cell-free filtrates, and their antibacterial, antifungal (against human and plant pathogens), and antioxidant properties were examined. Five promising fungi after screening were identified to possess all of these activities. These strains and their respective plant hosts were *Trichoderma harzianum* BUK-T (*Fagus sylvatica*), *Aspergillus ochraceus* ROB-L1 (*Robinia pseudoacacia*), *Chaetomium cochliodes* KLON-L1, *Fusarium tricinctum* KLON-L2 (*Acer platanoides*), and *Penicillium chrysogenum* SOS-B2 (*Pinus sylvestris*). Moreover, gamma irradiation at several doses (Gy) was separately applied to the fungal cultures to study their effects on the recorded activities. Finally, compounds after preparative thin-layer chromatography fractionation of the five fungal strains were identified by GC-MS. These findings suggest that the isolated endophytic fungi could serve as novel sources of bioactive metabolites with antibacterial, antifungal, and antioxidant properties, potentially paving the way for future research and the development of new bioactive compounds.

## 1. Introduction

The World Health Organisation reports accelerated rates of antimicrobial drug resistance in bacteria and fungi. With increasing multidrug resistance and the lack of many therapeutic agents previously applicable [1], novel bioactive compounds are becoming a promising solution. Moreover, identifying new sources and developing new natural antioxidants with innovative mode of actions represents a great challenge for the food industry. Several reports have proven the potential of plants as a source of natural bioactive compounds with antioxidant activity [2]. Indeed, the broad-spectrum potential of bioactive compounds from plants is well-known; however, the use of plants as sources of bioactive compounds may not be feasible considering the low yield, the difficulty in compound recovery, and their long maturation time [2]. It is necessary to alleviate our dependence on plants and to explore other environment-friendly alternatives for the production of these bioactive compounds, such as endophytic fungi of forest plants [3]. Such endophytes have proved to be promising sources of bioactive metabolites, thereby helping in the development of natural antimicrobials and antioxidants [2,3].

Generally, fungal endophytes are beneficial microbes that live inside healthy plant tissues [4]. They produce a wide variety of bioactive compounds belonging to diverse chemical classes and structures, encompassing alkaloids, flavonoids, steroids, saponins, glycosides, terpenoids, and phenolic compounds [5]. In addition, by using fermenters with controlled conditions for large-scale production, it is possible to achieve continuous, endless production of secondary metabolites, which, together with the rapid growth of mycelial biomass, makes endophytes a more profitable source than plants [6]. 

Being a valuable microbial resource, endophytic fungi are highly biodiverse and widely distributed throughout the natural world [7]. They are found in both terrestrial and aquatic plants, having been isolated from a diverse range of plants, such as bryophytes, ferns, horsetails, gymnosperms, and angiosperms, across various climates from tropical to arctic [8]. They populate plants from roots to shoots by bark, branches, leaves, and shoots to flowers, seeds, and fruits [9]. Moreover, the endophyte biodiversity is fascinating in its ubiquity. According to Xingyuan et al. [10] and Hardoim et al. [11], even from a single plant individual, tens of different fungi can be isolated, creating unique microecosystems, unrepeatable even in a plant individual of the same species. Also, endophytic fungi can produce bioactive compounds from slow-growing or rare and endangered plants [6,12]. For example, endophytic strains of *Aspergillus flavus*, *Alternaria alternate*, and *Aspergillus terreus* isolated from the stems of *Delonix regia*, *Tecoma stans*, and *Ricinus communis*, respectively, showed antifungal, antioxidant properties as well as seed germination promoting potentials [12]. In addition, several activities such as antidiabetic (*Alternaria* spp. isolated from *Viscum album*), antiparasitic and antimalarial (*Diaporthe miriciae*), and immunosuppressive (the genera *Byssochlamys*, *Penicillium*, *Septoria*, and *Aspergillus*) were reported [2].

Over the years, endophytic fungi and their metabolites have garnered significant interest due to their bioactive properties namely, inhibiting bacterial and fungal growth; modulation of the immune system; showing potential in cancer treatment; antioxidant, antidiabetic, antimalarial and antiviral effects; addressing parasitic infections; and acting as insect repellents [13]. They have proved to be untapped pools of novel bioactive molecules [14]. Hence, endophytic fungi are the subject of extensive research in medicine, pharmacy, agriculture, food, cosmetics, and various other disciplines [14,15]. In this regard, 43 fungal endophytes from the Mokrzański forest in Wrocław, Poland, were isolated. This paper aimed to bioprospect these endophytes as sources of antimicrobials and antioxidants. The impact of gamma irradiation on these activities was also investigated. Moreover, GC-MS analysis was conducted to identify the chemical constituents in the fungal extracts.

## 2. Results and Discussion

### 2.1. Screening Fungal Extracts for Antibacterial and Antifungal Activities

The antimicrobial potential of the fungal extracts (biomass and cell-free filtrate) was tested using the agar-well diffusion assay against human bacterial pathogens. The data presented in Table 1 show that biomass extracts from several fungal isolates showed antibacterial and antifungal activities. However, biomass extracts from cultures of three isolates with the code numbers 3 (isolated from the twigs of *Fagus sylvatica*), 4 (isolated from the leaves of *Robinia pseudoacacia*), and 43 (isolated from the bark of *Pinus sylvestris*) showed the three best activities. The recorded antimicrobial potentials of fungus no. 3 were 18.67 ± 0.58 (against *E. coli*), 18.00 ± 1.00 (against *S. aureus*), 15.67 ± 1.53 (against *A. brasiliensis*), 14.67 ± 0.58 (against *F. oxysporum*), and 16.67 ± 0.58 mm (against *C. albicans*). The recorded values of fungus no. 4 against the respective pathogens were 19.00 ± 1.00, 16.00 ± 1.00, 13.00 ± 0.58, 12.00 ± 1.53, and 15.33 ± 0.58 mm. In the case of fungus no. 43, these values were 15.00 ± 1.00, 14.67 ± 0.58, 11.33 ± 1.00, 12.00 ± 1.00, and 15.00 ± 1.00 mm, respectively. The data in Table 2 indicate that cell-free filtrate extracts from several fungal isolates showed the tested activities and two isolates named 7 and 42 (isolated from the leaves of *Acer platanoides*) had antibacterial and antifungal activities. The recorded antimicrobial potentials of the fungus no. 7 were 19.00 ± 1.00 (against *E. coli*), 18.33 ± 0.58 (against *S. aureus*), 12.00 ± 1.00 (against *A. brasiliensis*), 10.33 ± 0.58 (against *F. oxysporum*), and 15.67 ± 1.53 mm (against *C. albicans*). The recorded values of the fungus no. 42 against the respective pathogens were 20.00 ± 1.00, 18.33 ± 0.55, 10.00 ± 1.00, 12.67 ± 1.58, and 17.00 ± 1.00 mm. 

In the literature, several fungal strains isolated from *Pinus sylvestris* exhibited diverse bioactivities. For example, studies of the activity of the endophytic fungus *Talaromyces purpureogenus* isolated from leaves of *Pinus densiflora* showed that it significantly inhibited the growth of Gram-positive or Gram-negative pathogens [16]. Furthermore, another study detected the presence of flavonoids, alkaloids, phenols, saponins, steroids, tannins, and terpenoids in crude fungal extracts isolated from *Pinus roxburghii* [17]. This may explain the antimicrobial effect of endophytes of the *Pinus* genus. Generally, the antimicrobial effect of plant extracts of these species has previously been reported in the literature. For instance, extracts from *F. sylvatica* were active against *S. aureus*, including methicillin-resistant strains [18], especially against *E. coli* and *C. albicans* [19]. *Pezicula livida*, an endophytic fungus isolated from *F. sylvatica*, revealed an antimicrobial effect as a host by inhibition against the bacteria *Bacillus megaterium* and *E. coli* and the fungi *Ustilago violacea* and *Eurotium repens* [20]. In studies on *Acer* sp., *Betula* sp., and *Robinia* sp. plant extracts there is evidence of antibacterial and antifungal properties, which may be associated with rich polyphenol content [21,22,23,24], but there is no information as to the activity of the endophytic fungi residing in these plants.

### 2.2. Screening Fungal Extracts for Antioxidant Potential

Testing the antioxidant potential of the endophytic fungal extracts (either from biomass or from the cell-free filtrate) confirmed their capacity to combat free radicals. The obtained data confirmed a broad spectrum of the antioxidant activity of the extracts (Table 3). The obtained data further showed that both the biomass and the cell-free extracts from several fungal isolates showed DPPH scavenging potential with varying percentages. The highest values of the DPPH scavenging activity were obtained from biomass extract of fungus no. 3 (54.84 ± 10.73%), cell-free filtrate extract of fungus no. 20 (54.37 ± 1.48%), cell-free filtrate extract of fungus no. 42 (46.46 ± 11.91%), and biomass extract of fungus no. 43 (45.65 ± 3.67%). In general, antioxidant compounds are known to have anti-atherosclerotic, anticancer, anti-inflammatory, antibacterial, antiviral, and anticarcinogenic effects [25]. In addition, such antioxidants can be used to control deficits resulting from reactive oxygen species. In concurrence with our results, a previous report concluded that all fungal endophytes have some levels of antioxidant properties [26]. It has also been discovered that the wood and bark have a number of different polyphenols, and, despite this, flavonoids, hydroxycinnamic and procyanidins, and glycosylated saponins could be obtained from the leaves [27,28]. In previous studies, DPPH assays demonstrated a scavenging capacity of alcohol extracts and found the highest activity shown by samples containing higher concentrations of phenols [29]. It can be supposed that the nine endophytes isolated from *F. sylvatica* gained their activity in interactions with the host. Likewise, *Sorbus* sp. extracts owe their strong DPPH scavenging ability to terpenoids [30] as well as *Betula* sp. to a triterpene called betulin [31]. Previously, several endophytic fungi were reported to have antioxidant activities. For example, endophytic fungi isolated from the ethnomedicinal plant *Dillenia indica* L. [32] demonstrated good antioxidant activity in the DPPH assay [33].

### 2.3. Identification of the Selected Fungal Strains

**Figure 1:**
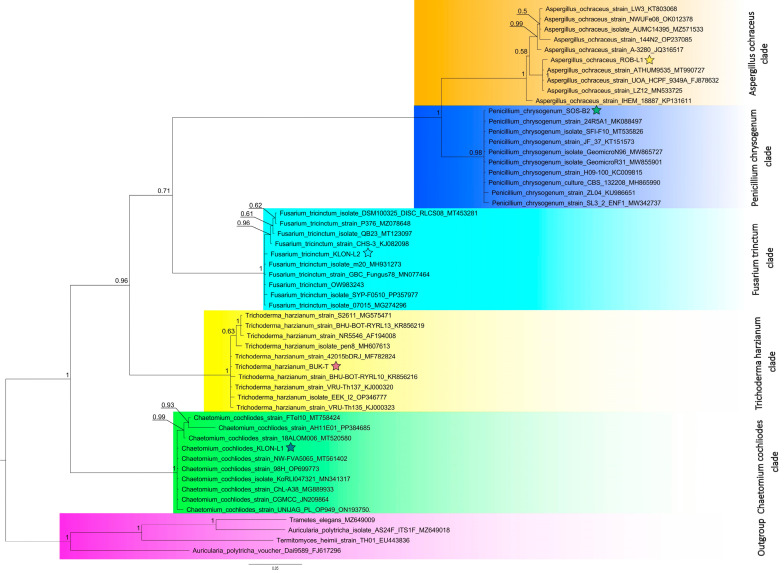
Bayesian phylogenetic tree of the ITS sequences of the five fungal isolates and sequences from NCBI. Numbers along the nodes are the posterior probabilities of the nodes. All distinct clades are shaded with different colors and separately described and all samples analyzed in this study are marked with a star.

Based on their promising activities, we selected the fungal strains with code nos. 3, 4, 7, 42, and 43 for molecular identification. After sequencing and data trimming, we collected at least 1000 copies for each barcoded sample (thus the coverage for each sample was at least 1000×). All sequenced and identified DNA samples were deposited in the NCBI database under accession numbers PP893016–PP893020. Identified species grouped with other representatives of the same genera on the tree (Figure 1) with high probability, which confirms their proper identification. Accordingly, the strains with code nos. 3, 4, 7, 42, and 43 are *Trichoderma harzianum* BUK-T, *Aspergillus ochraceus* ROB-L1, *Chaetomium cochliodes* KLON-L1, *Fusarium tricinctum* KLON-L2, and *Penicillium chrysogenum* SOS-B2, respectively.

### 2.4. Gamma Irradiation Effect on Growth and the Antibacterial, Antifungal, and Antioxidant Activities

**Figure 2:**
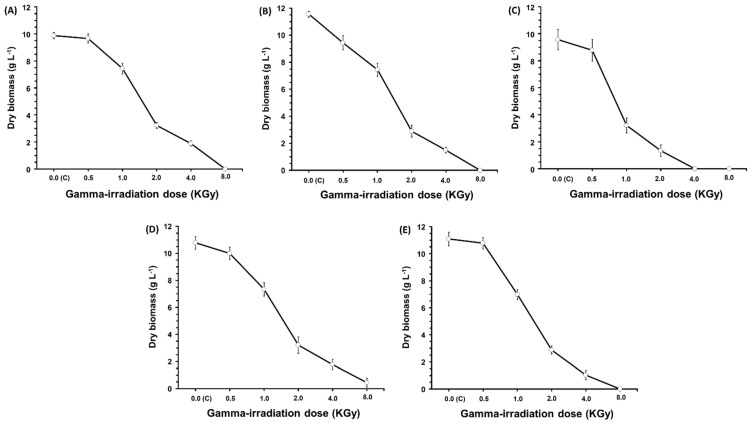
Effect of gamma irradiation of fungal growth of *Trichoderma harzianum* BUK-T (**A**), *Aspergillus ochraceus* ROB−L1 (**B**), *Chaetomium cochliodes* KLON−L1 (**C**), *Fusarium tricinctum* KLON−L2 (**D**), and *Penicillium chrysogenum* SOS−B2 (**E**).

Several doses ranging from 500 to 8000 Gy of gamma irradiation were used to study their impact on the antibacterial, antifungal, and antioxidant activities of extracts from *Trichoderma harzianum* BUK-T (biomass), *Aspergillus ochraceus* ROB-L1 (biomass), *Chaetomium cochliodes* KLON-L1 (cell-free filtrate), *Fusarium tricinctum* KLON-L2 (cell-free filtrate), and *Penicillium chrysogenum* SOS-B2 (biomass). Generally, the remarkable feature is the enhancing effect of gamma irradiation on the recorded activities of antibacterial, antifungal, and antioxidant activities of extracts from the five fungal strains. However, the best dose varies from one fungal strain to another and from one tested activity to another. Significant differences (*p ≤* 0.05) in all five strains in all of the measured activities (Table 4) were detected when compared to the control (0.00 Gy). In the literature, gamma radiation is a highly potent form of ionizing energy [33,34]. When living cells are exposed to specific doses of this radiation, it can induce mutations through mechanisms such as DNA repair [35,36]. Recently, gamma ray-induced mutagenesis has been strongly recommended for enhancing microbial strains to boost their production capabilities [37,38,39]. Supporting our findings in this study, gamma rays at 1000 Gy were used to significantly enhance the production of the cardiac glycoside digoxin by *Epicoccum nigrum*, resulting in a five-fold increase compared to control cultures [40]. Additionally, a 500 Gy dose of gamma radiation intensified the production of the acetylcholinesterase inhibitor HupA by *Alternaria brassicae* [41]. Our data (Figure 2) further indicated the dose-dependent manner of the effect of gamma irradiation on the fungal growth. Moreover, the lethal doses vary according to the fungal strain: *Trichoderma harzianum* BUK-T (Figure 2A), *Aspergillus ochraceus* ROB-L1 (Figure 2B), *Chaetomium cochliodes* KLON-L1 (Figure 2C), *Fusarium tricinctum* KLON-L2 (Figure 2D), and *Penicillium chrysogenum* SOS-B2 (Figure 2E). Similarly, several reports observed a delayed or reduced effect following exposure to gamma radiation at several doses [42,43,44,45,46,47,48,49].

### 2.5. GC-MS Analysis

The fungal extracts of the five strains were subjected to preparative thin-layer chromatography (TLC). Separated bands from the TLC plates were tested again for antibacterial, antifungal, and antioxidant activities. The active bands from TLC were also analyzed by GC-MS. The obtained results from *Trichoderma harzianum* BUK-T (Figure S1), *Aspergillus ochraceus* ROB-L1 (Figure S2), *Chaetomium cochliodes* KLON-L1 (Figure S3), *Fusarium tricinctum* KLON-L2 (Figure S4), and *Penicillium chrysogenum* SOS-B2 (Figure S5) revealed the existence of several compounds. The recorded retention times and the detected compounds are listed in Table 5. Per the literature listed in Table 5, our results showed the existence of various well-known compounds with diverse bioactivities such as anticancer, antioxidant, anti-inflammatory, antifungal, hypocholesterolemic, and hepatoprotective effects [50].

## 3. Materials and Methods

### 3.1. Cultivation Conditions and Preparation of Fungal Crude Extracts

**Figure 3:**
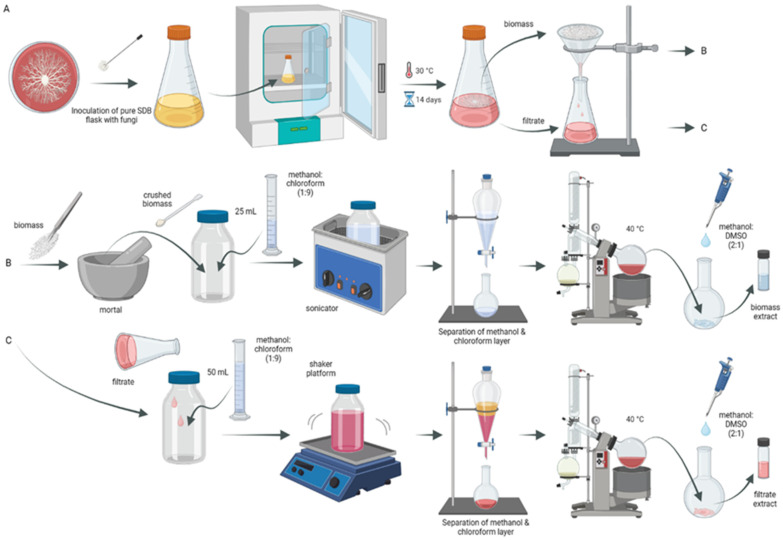
Schematic representation of the process of preparation of fungal extracts. (**A**) Cultivation of endophytic fungi and separation biomass from filtrate. (**B**) Biomass extract preparation by crushing, sonication, extraction, and concentration. (**C**) Filtrate extract preparation by shaking with solvents, extraction, and concentration. Created in BioRender.

Forty-three fungal strains were isolated from healthy plant parts collected from a local forest in Wrocław, Poland. For each fungal isolate, spore suspensions were prepared and adjusted to 10^5^ mL^−1^ using a hemocytometer from 7-day-old cultures. The prepared suspensions were used to inoculate Sabouraud Dextrose Broth (pH 6.0) medium (50 mL/250 mL Erlenmeyer flask) and incubated for 14 days under static conditions at 30 °C. After incubation, the cultures were filtered (through Whatman No. 1 filter paper) to separate the filtrate and fungus biomass. The filtrate of each fungus was extracted with a 50 mL mixture of CHCl_3_ and MeOH (9:1 *v*/*v*). Meanwhile, the biomasses were ground and crushed using mortar and pestle until they reached homogeneity. Then, 25 mL of a mixture of CHCl_3_ and MeOH (9:1 *v*/*v*) was added to each biomass homogenate. Extraction mixtures of the biomass were sonicated for 1 h in an ultrasonic bath (20 kHz, 20 °C), while the filtrate extraction mixtures were shaken overnight. After that, the chloroform–methanol layers were separated by a separating funnel, dehydrated by sodium sulfate anhydrous, and then concentrated by evaporation of the solvent on a vacuum evaporator. The resulting dry crude extract was dissolved in a mixture of MeOH and DMSO (2:1 *v*/*v*) and used for antimicrobial and antioxidant testing (Figure 3).

### 3.2. Antimicrobial Activity Screening

Antimicrobial potentials of the fungal extracts were evaluated using an agar-well diffusion assay [62]. Antibacterial sensitivity tests were carried out against human pathogenic strains *Staphylococcus aureus* ATCC6538 and *Escherichia coli* ATCC11229. The antifungal sensitivity tests were performed against one plant pathogen *Fusarium oxysporum* EUM37, and two human pathogens *Candida albicans* ATCC10231 and *Aspergillus brasiliensis* ATCC16404. The positive control for the antibacterial assay was a mixture of amoxicillin/clavulanic acid (500 µg/mL), that for the antifungal assay was nystatin (100 µg/mL), while the extract solvent (DMSO:MeOH = 1:2 *v*/*v*) was used as a negative control.

Bacterial suspensions were adjusted with a photometer at 600 nm to give a turbidity equivalent to the McFarland 0.5 standard (absorbance 0.08–0.1). Meanwhile, the fungal spore suspension was prepared and adjusted to 10^6^ spores per mL using a hemocytometer. Later, agar plates with Mueller–Hinton Agar (for bacteria), Potato-Dextrose Agar (for fungi), and Sabouraud Dextrose Agar (for *Candida*) were inoculated with 100 μL of the microorganism suspension. Agar wells were cut out and 50 μL of the fungal extract was placed into each well. Then, *E. coli*, *S. aureus*, and *C. albicans* plates were incubated at 37 °C, while *A. brasiliensis* and *F. oxysporum* plates were incubated at 30 °C for 24 h. Then, the incubation inhibition zones (ZOI) around the agar wells were measured.

### 3.3. DPPH Scavenging Activity Assay

DPPH (1,1-diphenyl-2-picryl hydrazyl) radical assays was used for the determination of the free radical-scavenging activities of the prepared extracts. To 120 μL of 100 µM DPPH methanol solution, 80 μL of extract or 10 mM ascorbic acid (as a positive control) or DMSO:MeOH (1:2 *v*/*v*) solvent as a blank were added. The absorbance was recorded at 517 nm after dark incubation for 15 min at room temperature [63]. The percentages of inhibition of DPPH were calculated:

Scavenging activity (%) = [(A_blank_ − A_sample_)/(A_blank_)] × 100

### 3.4. Fungal Strains

All 43 isolated endophytic fungi were tested as described earlier. Among them, five different isolates showed promising antimicrobial and antioxidant activities. These strains were *Trichoderma harzianum* BUK-T isolated from the twigs of *Fagus sylvatica*; *Chaetomium cochliodes* KLON-L1 and *Fusarium tricinctum* KLON-L2 isolated from the leaves of *Acer platanoides*; *Aspergillus ochraceus* ROB-L1 isolated from the leaves of *Robinia pseudoacacia*; and *Penicillium chrysogenum* SOS-B2 isolated from the bark of *Pinus sylvestris*.

### 3.5. Identification of the Selected Endophytic Fungi

Molecular characterization was used to identify the five strains. Cultures of the five selected fungal strains were used for DNA isolation with a Genomic Mini AX Yeast kit manufactured by A&A Biotechnology (Gdańsk, Poland) according to the producer’s manual. DNA isolation from all analyzed samples was carried out with a Genomic Mini AX Yeast kit manufactured by A&A Biotechnology (Gdańsk, Poland) according to the producer’s manual. The isolated DNA was analyzed with a Qubit 4.0 fluorimeter to specify the DNA quantity. Then, the ITS marker was amplified using ITS4/ITS5 primers as follows: initial denaturation at 95 °C—2 min; 35 cycles at 95 °C—60 s; 55 °C—60 s; 72 °C—90 s; and final elongation at 72 °C—10 min. The obtained PCR products were then verified using 1% agarose gel electrophoresis and cleaned using NucleoMag magnetic beads (Macherey-Nagel). Finally, all amplified and cleaned PCR products were sequenced using an Oxford Nanopore Technology MinION sequencer, using a SQK-NBD114.96 native barcoding kit and a FLO-MIN114 flow cell. The library preparation and sequencing processes were performed according to the ONT manuals for barcoded samples.

After sequencing, raw data (POD5 files) were base called using Dorado basecaller (https://github.com/nanoporetech/dorado, accessed on 1 January 2023) and a super accurate (SUP) model. Base called samples were analyzed for quality with FastQC (https://www.bioinformatics.babraham.ac.uk/projects/fastqc/, accessed on 1 January 2023) and trimmed accordingly (desired length and phred > 12) using chopper [64]. Finally, scaffolds for all samples were created with Scaffold Builder [65].

The Mycobank database was used to identify the species of all samples and all identified samples were used to prepare a phylogenetic tree presenting its taxonomic position. Apart from DNA samples revealed in this study, ITS fragments for all identified species were obtained from the NCBI database, along with five other species used as outgroups for rooting. Phylogeny was resolved with the Bayesian approach using MrBayes 3.2.7a software [66], and the SYM +G +I substitution model was chosen as the best fit for the data by jModelTest [67]. Trees were sampled every 100th MCMC generation for 6,000,000 generations until the average standard deviation of split frequencies was stabilized at a value much below 0.01 for all trees used to construct the consensus tree.

### 3.6. Effect of ^60^Co Gamma Irradiation on Growth and the Tested Activities

Spore suspensions of *Trichoderma harzianum* BUK-T, *Aspergillus ochraceus* ROB-L1, *Chaetomium cochliodes* KLON-L1, *Fusarium tricinctum* KLON-L2, and *Penicillium chrysogenum* SOS-B2 were prepared as described earlier and separately irradiated at 0.5, 1, 2, 4, and 8 kGy using a ^60^Co Gamma chamber with dose rate of 311.88 Gy h^−1^. The irradiated suspensions were kept in darkness overnight. The irradiated suspensions were used to inoculate flasks as mentioned before. After incubation, the fungal cultures were extracted and tested. To estimate the fungal growth (g L^−1^), fresh biomass was collected by filtration then dried to a constant weight (50 °C in a hot air oven).

### 3.7. Analytical Methods

#### 3.7.1. Thin-Layer Chromatography (TLC) Fractionation

The fungal extracts of *Trichoderma harzianum* BUK-T, *Aspergillus ochraceus* ROB-L1, *Chaetomium cochliodes* KLON-L1, *Fusarium tricinctum* KLON-L2, and *Penicillium chrysogenum* SOS-B2 (with the highest recorded activity potentials) were subjected to TLC fractionation (GF-254, Merck, Germany) using as the eluent chloroform:methanol (9:1, *v*/*v*). The developed TLC plates were air-dried and examined under a UV lamp. The separated bands were carefully removed by scraping off the silica gel then eluting the same mixture (methanol:DMSO, 2:1 *v*/*v*), and re-tested again for the studied activities (as described earlier).

#### 3.7.2. GC-MS Analysis

Active fractions from preparative TLC of *Trichoderma harzianum* BUK-T, *Aspergillus ochraceus* ROB-L1, *Chaetomium cochliodes* KLON-L1, *Fusarium tricinctum* KLON-L2, and *Penicillium chrysogenum* SOS-B2 were analyzed with a Shimadzu GCMS QP 2020 (Shimadzu, Kyoto, Japan) equipped with a 30 m × 0.25 mm × 0.25 µm ZB-5 column (Phenomenex, Torrance, CA, USA). The sample (1 µL) was injected at 260 °C with split 100 and helium with a linear velocity 35 cm·s^−1^ as a carrier gas. For the separation of analytes, the following temperature program was used: initial 50 °C to 250 °C at a rate of 3 °C·min^−1^. The mass spectrometer was configured for the SCAN mode with a range of 35 to 650 *m*/*z*; the interface temperature and the ion source temperature were 250 °C. Identification of potential analytes was based on the similarity of the mass spectra obtained experimentally with the mass spectra available in the NIST20 library (National Institute of Standards and Technology).

### 3.8. Statistics

Statistical analysis was based on determining the average values of triplicate measurements and their standard deviation, as well as one-way ANOVA (analysis of variation), using SPSS (V.22) software at the significance level *p* < 0.05.

## 4. Conclusions

Five different promising endophytic fungi, namely *Trichoderma harzianum* BUK-T, *Aspergillus ochraceus* ROB-L1, *Chaetomium cochliodes* KLON-L1, *Fusarium tricinctum* KLON-L2, and *Penicillium chrysogenum* SOS-B2 showed bioactive metabolites in their extracts with antimicrobial potential against certain human and plant pathogens as well as antioxidant properties in their extracts. Gamma irradiation was successfully applied to intensify the studied bioactivities. GC-MS was used to identify compounds of the five endophytic fungal strains after preparative thin-layer chromatography fractionation of their extracts. These findings suggest that bioprospecting endophytic fungi from forest plants could be a promising approach to discovering untapped sources of antimicrobials and antioxidants. The presented research also highlights the exploration of this unique environment, which can lead to bioactive compounds with potential medical, industrial, food, or agricultural applications and enrich scientific knowledge.

## Figures and Tables

**Table 1:** Antibacterial and antifungal activities of the biomass extracts from the isolated fungal culture.

Fungi Code No.	Diameter of Inhibition Zone (mm)				
	*E. coli*	*S. aureus*	*A. brasiliensis*	*F. oxysporum*	*C. albicans*
3	18.67 ± 0.58	18.00 ± 1.00	15.67 ± 1.53	14.67 ± 0.58	16.67 ± 0.58
4	19.00 ± 1.00	16.00 ± 1.00	13.00 ± 0.58	12.00 ± 1.53	15.33 ± 0.58
7	19.33 ± 1.53	17.67 ± 0.58	Nil	Nil	Nil
10	Nil	Nil	12.67 ± 1.53	11.33 ± 0.58	10.00 ± 1.00
12	Nil	Nil	Nil	Nil	9.67 ± 1.53
13	Nil	Nil	18.67 ± 0.58	10.67 ± 0.58	11.00 ± 1.00
15	13.00 ± 1.00	12.67.00 ± 0.58	Nil	Nil	Nil
17	Nil	Nil	Nil	Nil	10.00 ± 1.00
20	10.67 ± 1.58	11.00 ± 1.00	Nil	Nil	Nil
22	Nil	Nil	Nil	Nil	12.33 ± 0.55
24	10.33 ± 0.58	12.00 ± 1.00	Nil	Nil	Nil
27	Nil	Nil	10.00 ± 1.00	10.67 ± 1.58	15.33 ± 0.58
28	10.33 ± 0.58	12.00 ± 1.00	Nil	Nil	Nil
30	10.33 ± 0.58	12.00 ± 1.00	Nil	Nil	Nil
32	Nil	Nil	Nil	Nil	11.00 ± 1.00
34	14.67 ± 1.58	12.00 ± 1.00	Nil	Nil	Nil
35	Nil	Nil	Nil	Nil	Nil
37	12.33 ± 0.55	10.00 ± 1.00	Nil	Nil	Nil
39	Nil	Nil	Nil	Nil	14.33 ± 0.58
40	13.67 ± 1.58	14.33 ± 0.58	Nil	Nil	Nil
42	Nil	Nil	14.00 ± 1.00	11.67 ± 0.58	12.67 ± 0.58
43	15.00 ± 1.00	14.67 ± 0.58	11.33 ± 1.00	12.00 ± 1.00	15.00 ± 1.00
Control	9.00	9.00	11.00	10.00	12.00
LSD	0.875	0.619	0.557	0.398	0.661

Nil means that no ZOI (Zone of inhibition) was detected. Amoxicillin (antibacterial standard) and nystatin (antifungal standard) were the controls. The calculated mean is for triplicate measurements from three independent experiments ± SD, LSD = least significant differences (*p* ≤ 0.05).

**Table 2:** Antibacterial and antifungal activities of the cell-free filtrate extracts from the isolated fungal culture.

Fungi Code No.	Diameter of Inhibition Zone (mm)				
	*E. coli*	*S. aureus*	*A. brasiliensis*	*F. oxysporum*	*C. albicans*
3	10.33 ± 0.58	10.00 ± 1.00	Nil	Nil	Nil
4	12.00 ± 1.00	13.67 ± 1.58	Nil	Nil	Nil
5	Nil	Nil	Nil	Nil	10.00 ± 1.00
7	19.00 ± 1.00	18.33 ± 0.58	12.00 ± 1.00	10.33 ± 0.58	15.67 ± 1.53
10	Nil	Nil	10.00 ± 1.00	9.67 ± 1.58	12.33 ± 0.55
13	10.00 ± 1.00	11.33 ± 1.55	Nil	Nil	11.00 ± 1.00
15	14.33 ± 0.58	13.00 ± 1.00	Nil	Nil	Nil
17	Nil	Nil	Nil	Nil	12.00 ± 1.00
20	Nil	Nil	Nil	Nil	14.00 ± 1.00
22	13.00 ± 1.00	12.67 ± 1.58	Nil	Nil	Nil
23	Nil	Nil	Nil	Nil	9.33 ± 0.55
24	14.33 ± 0.58	16.00 ± 1.00	Nil	Nil	Nil
26	12.33 ± 0.55	11.67 ± 1.58	Nil	Nil	Nil
27	Nil	Nil	Nil	Nil	15.33 ± 0.58
28	14.00 ± 1.00	14.33 ± 0.55	Nil	Nil	Nil
30	11.67 ± 1.58	10.33 ± 0.55	Nil	Nil	Nil
31	Nil	Nil	15.00 ± 1.00	13.33 ± 0.58	Nil
32	Nil	Nil	Nil	Nil	12.00 ± 1.00
34	10.00 ± 1.00	11.33 ± 0.55	Nil	Nil	Nil
37	14.00 ± 1.00	12.67 ± 1.58	Nil	Nil	Nil
39	Nil	Nil	Nil	Nil	10.33 ± 0.58
40	10.33 ± 0.55	10.67 ± 1.58	Nil	Nil	Nil
42	20.00 ± 1.00	18.33 ± 0.55	10.00 ± 1.00	12.67 ± 1.58	17.00 ± 1.00
43	Nil	Nil	9.00 ± 1.00	10.33 ± 0.55	13.67 ± 1.58
Control	9.00	9.00	11.00	10.00	12.00
LSD	0.739	0.821	0.872	0.298	0.871

Nil means that no ZOI was detected. Amoxicillin (antibacterial standard) and nystatin (antifungal standard) were the controls. The calculated mean is for triplicate measurements from three independent experiments ± SD, LSD = least significant differences (*p* ≤ 0.05).

**Table 3:** Antioxidant activities of the biomass extract and cell-free filtrate extract from the isolated fungal culture.

Fungi Code No.	Scavenging Activity (%)	
	Biomass Extract	Cell-Free Filtrate Extract
1	Nil	21.76 ± 1.47
3	54.84 ± 10.73	Nil
4	44.96 ± 8.45	Nil
6	Nil	13.21 ± 2.56
7	67.41 ± 10.7	25.76 ± 5.87
8	12.91 ± 0.05	10.44 ± 1.36
9	21.93 ± 0.15	9.48 ± 1.05
10	12.09 ± 0.33	14.36 ± 1.58
15	Nil	62.47 ± 10.13
18	Nil	10.46 ± 0.34
20	34.55 ± 6.21	54.37 ± 1.48
22	4.87 ± 5.21	38.21 ± 11.46
23	20.77 ± 11.2	Nil
24	44.87 ± 9.21	25.57 ± 1.58
29	Nil	14.56 ± 1.48
30	7.90 ± 0.79	Nil
31	11.57 ± 1.45	27.58 ± 0.88
32	18.43 ± 1.21	12.00 ± 1.00
34	12.76 ± 1.05	43.65 ± 7.78
35	24.76 ± 1.89	26.01 ± 5.38
39	7.43 ± 0.17	13.57 ± 1.21
42	31.55 ± 2.32	46.46 ± 11.91
43	45.65 ± 3.67	Nil
Control	85.32	85.32
LSD	5.985	6.921

Nil means that no activity was detected. Ascorbic acid (antioxidant standard) was the control. The calculated mean is for triplicate measurements from three independent experiments ± SD, LSD = least significant differences (*p* ≤ 0.05).

**Table 4:** Effect of gamma irradiation on the antibacterial, antifungal, and antioxidant activities of extracts from *Trichoderma harzianum* BUK-T (biomass), *Aspergillus ochraceus* ROB-L1 (biomass), *Chaetomium cochliodes* KLON-L1 (cell-free filtrate), *Fusarium tricinctum* KLON-L2 (cell-free filtrate), and *Penicillium chrysogenum* SOS-B2 (biomass).

Fungal Strain	Dose (kGy)	Diameter of Inhibition Zone (mm)					Scavenging Activity (%)
		*E. coli*	*S. aureus*	*A. brasiliensis*	*F. oxysporum*	*C. albicans*	
*T. harzianum* BUK-T	0.0 (C)	18.00 ± 1.00	17.67 ± 1.58	14.33 ± 0.55	12.00 ± 1.00	16.33 ± 0.55	55.02 ± 9.89
	0.5	20.33 ± 0.55	19.00 ± 1.00	14.00 ± 1.00	14.33 ± 0.55	18.00 ± 1.00	58.43 ± 7.31
	1	22.67 ± 1.67	24.00 ± 1.00	16.67 ± 1.58	14.67 ± 1.58	21.00 ± 1.0	75.32 ± 10.03
	2	10.00 ± 1.00	9.33 ± 0.55	10.00 ± 1.00	9.33 ± 0.55	12.67 ± 1.58	43.76 ± 7.55
	4	Nil	Nil	Nil	Nil	Nil	10.32 ± 1.76
	8	Nil	Nil	Nil	Nil	Nil	Nil
*A. ochraceus* ROB-L1	0.0 (C)	19.33 ± 0.55	15.67 ± 1.58	14.00 ± 1.00	12.33 ± 0.55	14.67 ± 1.58	45.06 ± 10.21
	0.5	19.67 ± 1.58	16.00 ± 1.00	14.67 ± 1.58	14.00 ± 1.00	15.33 ± 0.55	47.91 ± 11.45
	1	20.00 ± 1.00	18.33 ± 0.55	16.00 ± 1.00	15.67 ± 1.58	18.00 ± 1.00	56.97 ± 5.81
	2	23.00 ± 1.00	25.00 ± 1.00	18.67 ± 1.58	17.00 ± 1.00	23.33 ± 0.55	67.37 ± 12.41
	4	10.67 ± 1.58	11.33 ± 0.55	12.33 ± 0.55	11.67 ± 1.58	10.00 ± 1.00	32.90 ± 7.38
	8	Nil	Nil	Nil	Nil	Nil	Nil
*C. cochliodes* KLON-L1	0.0 (C)	18.67 ± 1.58	17.67 ± 1.58	12.33 ± 0.55	11.00 ± 1.00	14.33 ± 0.55	26.41 ± 8.91
	0.5	21.00 ± 1.00	19.33 ± 0.55	14.00 ± 1.00	14.33 ± 0.55	23.00 ± 1.00	31.44 ± 5.89
	1	19.33 ± 0.55	16.00 ± 1.00	10.67 ± 1.58	12.00 ± 1.00	16.33 ± 0.55	22.19 ± 10.34
	2	10.66 ± 1.58	9.00 ± 1.00	9.33 ± 0.55	10.67 ± 1.58	9.00 ± 1.00	6.37 ± 1.28
	4	Nil	Nil	Nil	Nil	Nil	Nil
	8	Nil	Nil	Nil	Nil	Nil	Nil
*F. tricinctum* KLON-L2	0.0 (C)	19.67 ± 1.58	18.00 ± 1.00	10.33 ± 0.55	11.33 ± 0.55	16.67 ± 1.58	45.81 ± 10.52
	0.5	19.33 ± 0.55	19.00 ± 1.00	12.00 ± 1.00	13.00 ± 1.00	18.33 ± 0.55	61.32 ± 12.31
	1	26.00 ± 1.00	25.67 ± 1.58	16.00 ± 1.00	15.67 ± 1.58	24.00 ± 1.00	87.51 ± 14.02
	2	20.33 ± 0.55	21.00 ± 1.00	14.33 ± 0.55	14.00 ± 1.00	20.33 ± 0.55	77.37 ± 12.43
	4	9.00 ± 1.00	10.33 ± 0.58	11.67 ± 1.58	9.00 ± 1.00	10.00 ± 1.00	Nil
	8	Nil	Nil	Nil	Nil	Nil	Nil
*P. chrysogenum* SOS-B2	0.0 (C)	14.00 ± 1.00	14.33 ± 0.55	10.67 ± 1.58	11.67 ± 1.58	14.67 ± 1.58	44.98 ± 7.31
	0.5	14.00 ± 1.00	15.00 ± 1.00	12.00 ± 1.00	12.33 ± 0.55	15.00 ± 1.00	67.32 ± 12.41
	1	16.67 ± 1.58	16.67 ± 1.58	13.00 ± 1.00	14.00 ± 1.00	15.33 ± 0.55	71.22 ± 14.57
	2	20.00 ± 1.0	19.00 ± 1.00	18.67 ± 1.58	14.67 ± 1.58	18.00 ± 1.00	73.49 ± 10.21
	4	Nil	Nil	Nil	Nil	Nil	3.67 ± 1.22
	8	Nil	Nil	Nil	Nil	Nil	Nil
Control		9.00	9.00	11.00	10.00	12.00	85.32
LSD		0.840	0.872	0.698	0.871	0.569	6.921

Nil means that no ZOI was detected. Amoxicillin (antibacterial standard), nystatin (antifungal standard), and ascorbic acid (antioxidant standard) were used as the controls. The calculated mean is for triplicate measurements from three independent experiments ± SD, LSD = least significant differences (LSD test, *p* ≤ 0.05).

**Table 5:** GC-MS analysis of active bands of active fractions of extracts from *Trichoderma harzianum* BUK-T (biomass), *Aspergillus ochraceus* ROB-L1 (biomass), *Chaetomium cochliodes* KLON-L1 (cell-free filtrate), *Fusarium tricinctum* KLON-L2 (cell-free filtrate), and *Penicillium chrysogenum* SOS-B2 (biomass).

Fungus	S.N.	RT (min)	Detected Compounds	Bioactivity	Reference
*T. harzianum* BUK-T	1	41.19	Pyrrolo[1,2-a]pyrazine-1,4-dione, hexahydro-	Anticancer, antioxidant	[50]
	2	47.12	Palmitic acid	Anti-inflammatory	[51]
	3	53.7	*cis*-9-Hexadecenal	Antifungal, antimelanogenic	[52]
	4	54.4	Stearic acid	Neuroprotection	[53]
	5	59.97	9-Octadecenamide, (*Z*)-	Lipid metabolism	[54]
*A. ochraceus* ROB-L1	1	43.351	Cyclo(leucyloprolyl) derivative		
	2	46.523	Cyclo(leucyloprolyl)	Antimicrobial	[55]
	3	47.078	Cyclo(leucyloprolyl) derivative		
	4	60.33	Pyrrolo[1,2-a]pyrazine-1,4-dione, hexahydro-3-(phenylmethyl)-	Anticancer	[50]
	5	66.7	unknown		
*C. cochliodes* KLON-L1	1	39.305	7-Oxabicyclo[4.1.0]heptane, 3-oxiranyl-		
	2	39.614	Uric acid	Antioxidant	[56]
	3	43.351	Cyclo(leucyloprolyl) derivative		
	4	44.36	1-(3-Methylbutyryl)pyrrolidine		
	5	46.523	Cyclo(leucyloprolyl)	Antimicrobial	[55]
	6	47.078	Cyclo(leucyloprolyl) derivative		
	7	47.313	Proline		
	8	47.489	Cyclo(leucyloprolyl) derivative		
	9	48.226	Palmitic acid	Anti-inflammatory	[51]
	10	56.264	Cyclo(leucyloprolyl) derivative		
	11	58.995	Dehydroergotamine	Antimicrobial	[57]
	12	59.966	9-Octadecenamide, (*Z*)-	Lipid metabolism	[54]
	13	60.33	Pyrrolo[1,2-a]pyrazine-1,4-dione, hexahydro-3-(phenylmethyl)-	Anticancer	[50]
*F. tricinctum* KLON-L2	1	59.29	Squalene	Antioxidant, anticancer	[58]
	2	64.9	Octacosane, 2-methyl-		
*P. chrysogenum* SOS-B2	1	41	Pentadecanoic acid		
	2	48.7	Linolelaidic acid, methyl ester		
	3	50.51	9,12-Octadecadien-1-ol, (*Z*,*Z*)	Anti-inflammatory, hypocholesterolemic, hepatoprotective	[59]
	4	50.7	Oleic Acid	Antifungal, antitermite	[60]
	5	51.86	Behenic acid	Antibacterial	[61]
	6	58.84	Oleic acid amide	Antifungal, antitermite	[60]

## Data Availability

All data generated or analyzed during this study are included in this published article.

## Supplementary Materials

The following supporting information can be downloaded at: https://www.mdpi.com/article/10.3390/molecules29194746/s1, Figure S1: GC-MS chromatograms of the detected compounds from *Trichoderma harzianum* BUK-T; Figure S2: GC-MS chromatograms of the detected compounds from *Aspergillus ochraceus* ROB-L1; Figure S3: GC-MS chromatograms of the detected compounds from *Chaetomium cochliodes* KLON-L1; Figure S4: GC-MS chromatograms of the detected compounds from *Fusarium tricinctum* KLON-L2; Figure S5: GC-MS chromatograms of the detected compounds from *Penicillium chrysogenum* SOS-B2.
