# Supplementary material for: Bioprospecting Endophytic Fungi of Forest Plants for Bioactive Metabolites with Antibacterial, Antifungal, and Antioxidant Potentials
Source: Molecules. 2024 Oct 8;29(19):4746. doi: 10.3390/molecules29194746 (PMC11477511; doi:10.3390/molecules29194746)
Supplement: Supplementary file 1 [file molecules-29-04746-s001.zip › molecules-3211764-supplementary.pdf]

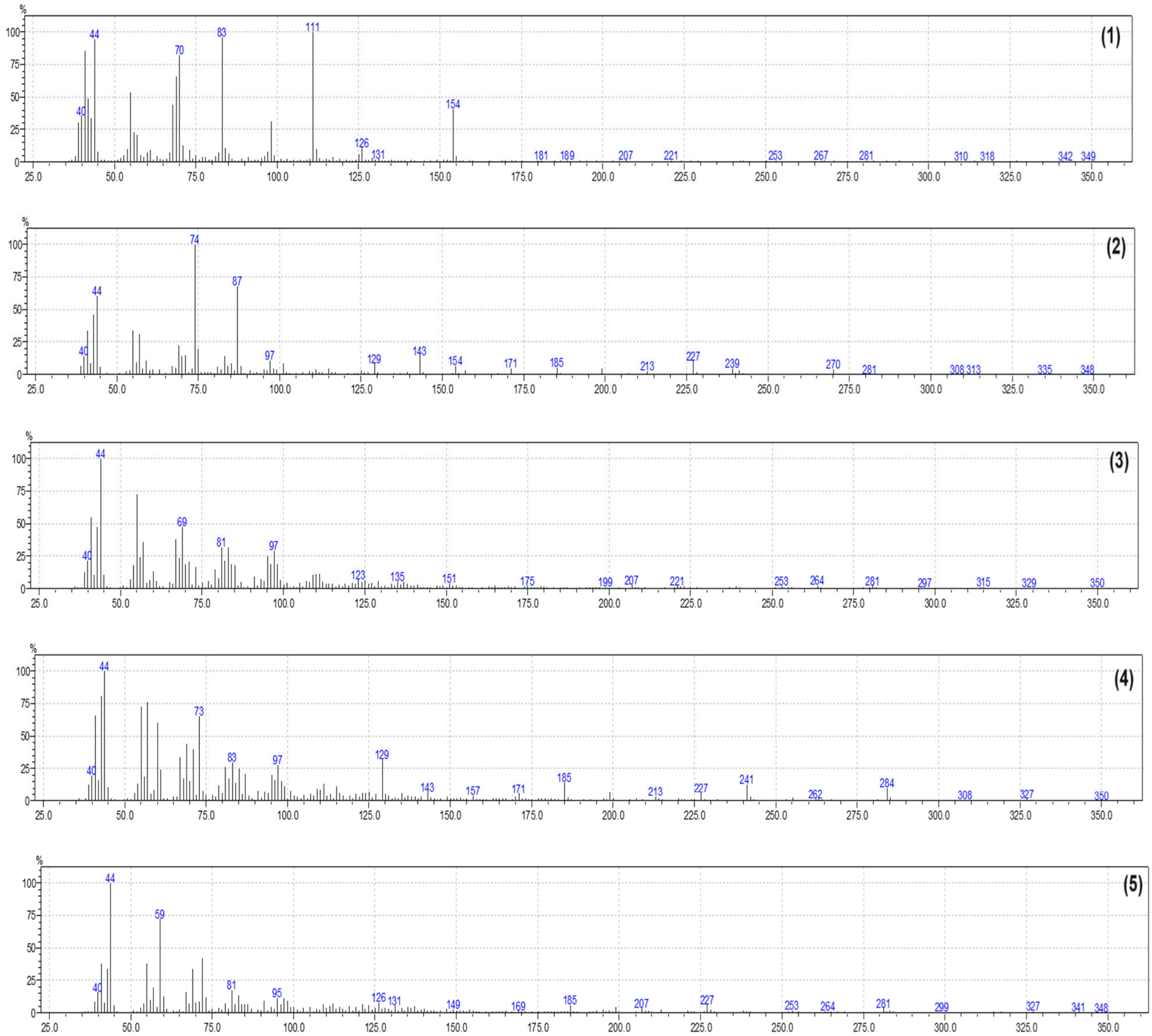

Figure S1 GC-MS chromatograms of the detected compounds (names corresponding to numbers are listed in Table 5) from *Trichoderma harzianum* BUK-T.

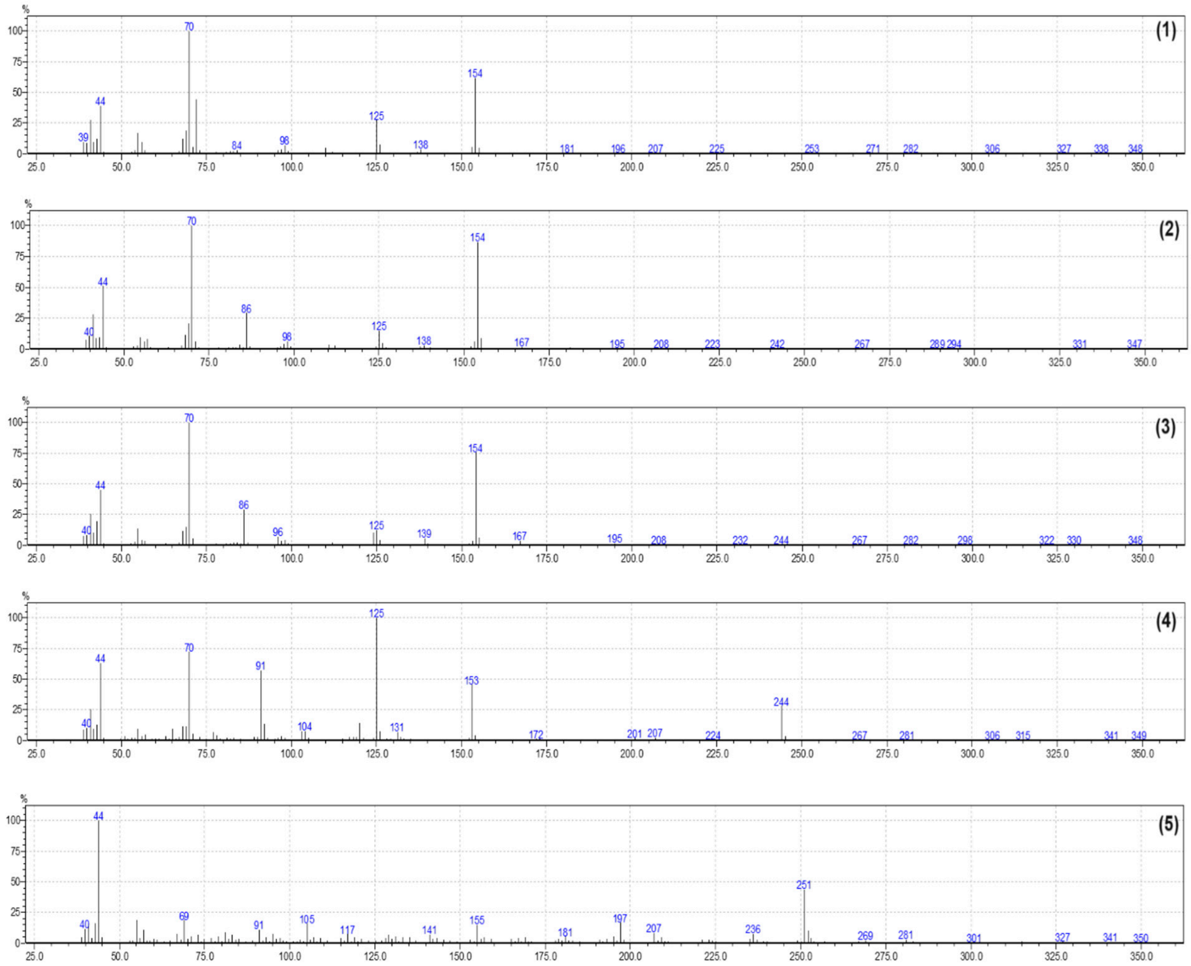

Figure S2 GC-MS chromatograms of the detected compounds (names corresponding to numbers are listed in Table 5) from *Aspergillus ochraceus* ROB-L1;

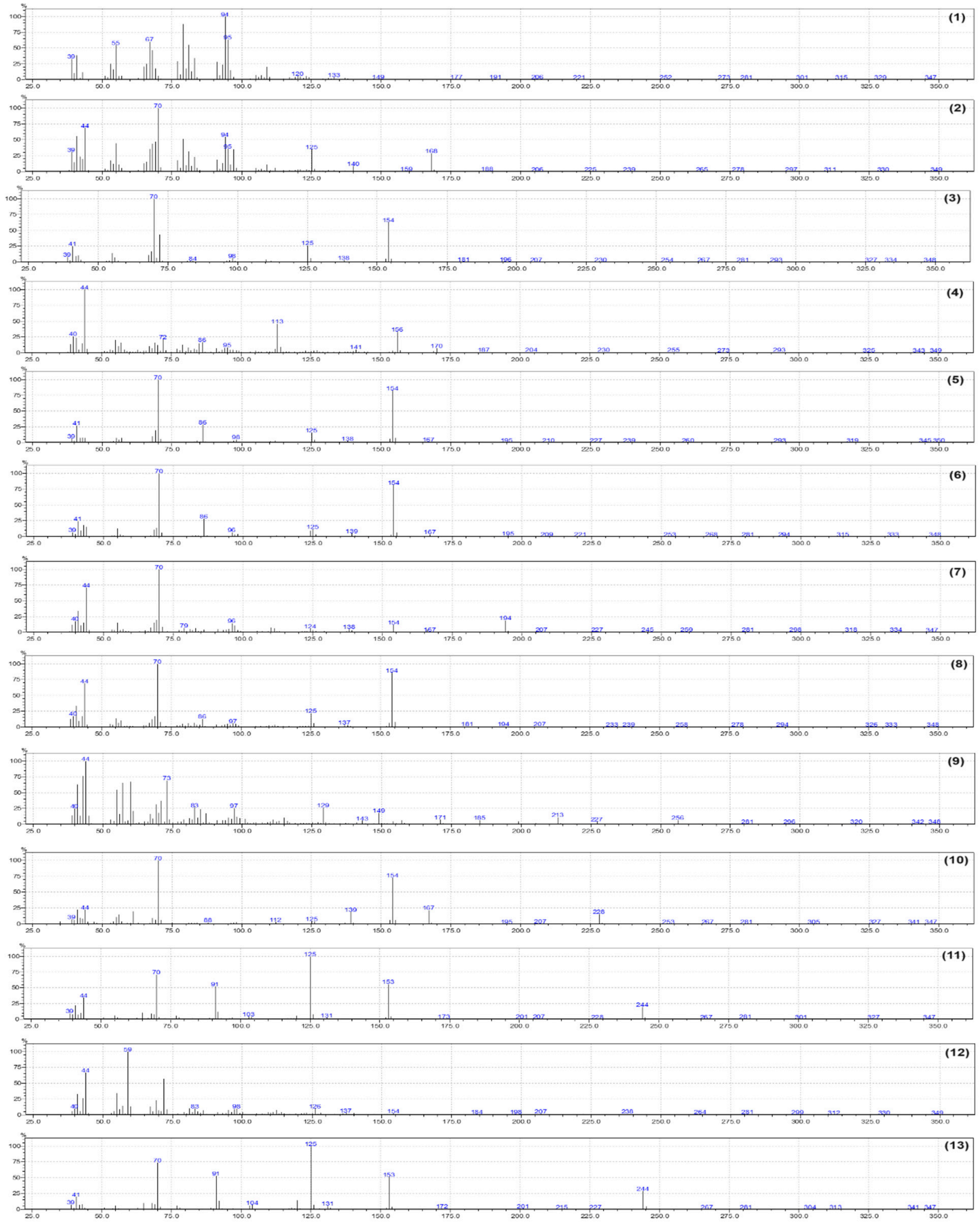

Figure S3 GC-MS chromatograms of the detected compounds (names corresponding to numbers are listed in Table 5) from *Chaetomium cochliodes* KLON-L1;

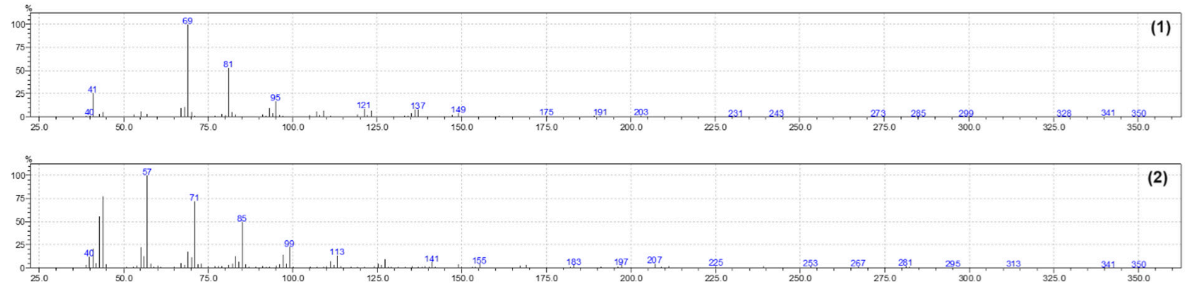

Figure S4 GC-MS chromatograms of the detected compounds (names corresponding to numbers are listed in Table 5) from *Fusarium tricinctum* KLON-L2;

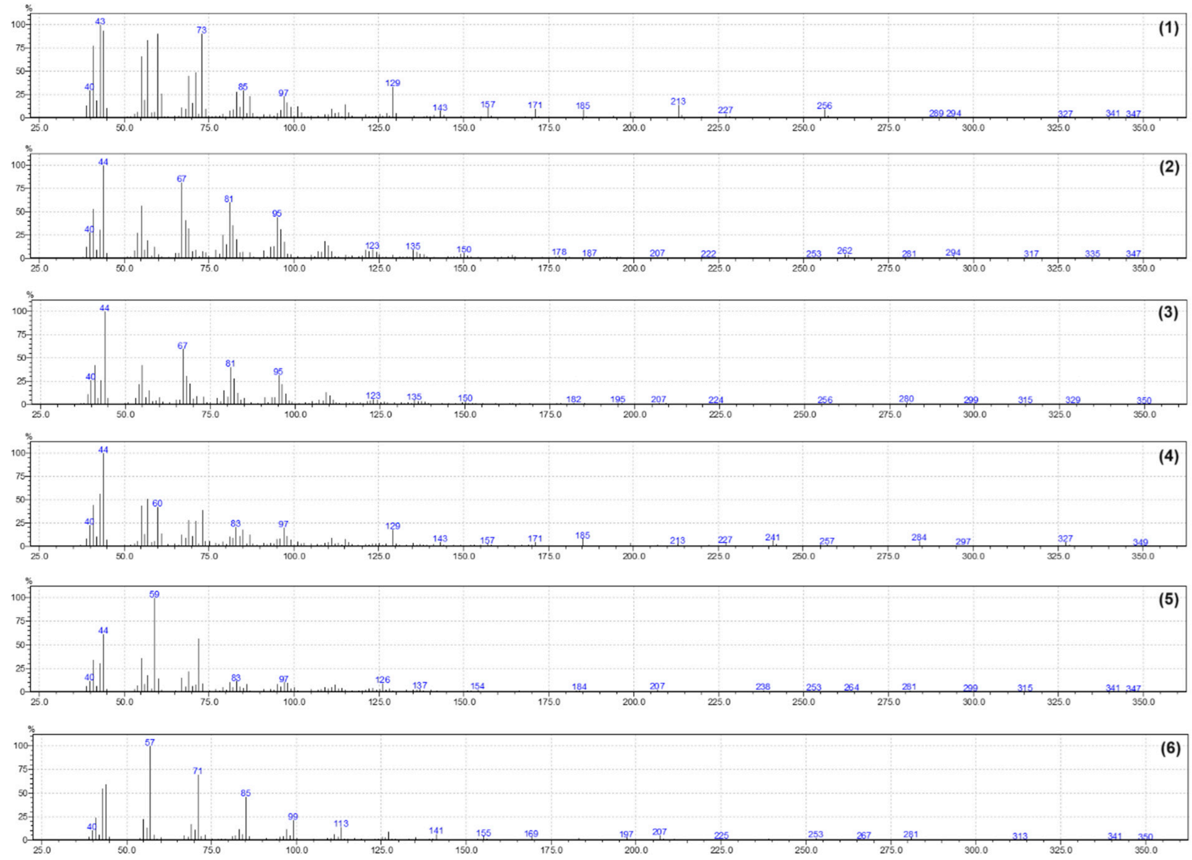

Figure S5 GC-MS chromatograms of the detected compounds (names corresponding to numbers are listed in Table 5) from *Penicillium chrysogenum* SOS-B2.
